# Examining Gender Differences and the Impact of Additional Fourth Year Rotations on Emergency Medicine Standardized Letter of Evaluation (SLOE) Scores

**DOI:** 10.7759/cureus.94488

**Published:** 2025-10-13

**Authors:** Abagayle Bierowski, Zaid Tayyem, Casey Morrone, Erin Hoag, Kelly Kehm, Carlos Rodriguez, Chaiya Laoteppitaks, Peter J Tomaselli, Dimitrios Papanagnou, Xiao Chi Zhang

**Affiliations:** 1 Emergency Medicine, Thomas Jefferson University, Philadelphia, USA; 2 Emergency Medicine, Thomas Jefferson University-Abington Hospital, Abington, USA; 3 Emergency Medicine, Wake Forest University, Winston-Salem, USA; 4 Emergency Medicine, Thomas Jefferson University Hospital, Philadelphia, USA

**Keywords:** gender comparison, residency application, rotation quantity, sloe, sub-internship

## Abstract

Introduction: The Emergency Medicine (EM) Standardized Letter of Evaluation (SLOE) is a critical component of the residency application process, yet questions remain regarding potential gender bias and the impact of repeated fourth-year rotations on applicant scores. This study examined gender differences in SLOE ratings and evaluated whether completing additional rotations influenced scores.

Methods: A retrospective review of 243 SLOEs from 101 residents at a large urban academic EM residency program (2015-2021) was conducted, excluding non-EM evaluations and incomplete forms. SLOE items were converted to numerical scores for analysis. Independent-sample t-tests assessed gender differences across 10 quantitative questions, while one-way ANOVA evaluated differences in scores across first, second, and third sub-I rotations.

Results: Female applicants received higher scores than male applicants on all 10 items, with statistically significant differences in four domains: ability to formulate a differential/treatment plan (2.41 vs. 2.20, p=0.008), ability to communicate a caring nature to patients (2.64 vs. 2.48, p=0.029), overall rotation grade (2.41 vs. 2.20, p=0.015), and estimated rank list position (2.78 vs. 2.54, p=0.04). No significant gender differences were observed in commitment to EM, teamwork, work ethic, or predicted success in residency. Comparison of fourth-year medical students' first, second, and third EM rotations (n=159) revealed no statistically significant differences across SLOE scores (p>0.05), although trends suggested the strongest overall performance during the first rotation.

Conclusion: Female applicants received significantly higher ratings in select evaluative domains, consistent with prior research on gender-related variability in SLOEs. Additional rotations did not yield statistically significant improvements in scores, supporting current guidance that applicants should not complete more than two EM fourth-year rotations. These findings underscore the potential influence of home institution dynamics on early SLOE evaluations and support the need for greater standardization and transparency in evaluation practices to promote fairness and equity in the residency selection process.

## Introduction

The Emergency Medicine (EM) Standardized Letter of Evaluation (SLOE) is a crucial assessment tool used to evaluate EM residency applicants during their fourth-year rotations [1]. The SLOE includes both quantitative and qualitative elements, incorporating numerical ratings across domains such as clinical skills, work ethic, and interpersonal communication; global assessments comparing applicants to their peers; and narrative comments highlighting strengths, areas for growth, and overall impressions [2-4]. Together, these components provide program directors with a standardized, comparative analysis of an applicant’s performance and competitiveness. Despite its critical role in the selection process, the SLOE's predictive validity and potential biases, particularly regarding gender differences and the impact of repeated sub-internship rotations, remain underexplored.

Several studies have investigated potential gender biases in the language used in SLOEs and gender differences in performance on specific SLOE measures; evaluators tend to describe female applicants using more communal and social descriptors, whereas male applicants are more often characterized with agentic, achievement-oriented terminology [5-7]. Andrusaitis et al. (2020) reported that female applicants performed better than male applicants on the comparative rank score, global assessment score, and anticipated rank list position in SLOEs [8]. Similarly, Mannix et al. (2022) determined that female applicants scored higher than male applicants on the qualifications for EM, global assessment, and anticipated rank list position, though certain qualifications for EM were weighed differently for men and women [9]. Other work has demonstrated clear racial bias in EM SLOEs, with inequities persisting even after controlling for performance predictors and differences in descriptive language used for underrepresented in medicine (URiM) applicants that may impact perceived hireability; URiM is typically defined as racial and ethnic populations that are underrepresented in the medical profession relative to their numbers in the general population, most commonly including Black, Hispanic/Latino, and Native American individuals [10,11]. These disparities have been observed both in the form of lower global assessment ratings and in narrative descriptors that emphasize personal background or communal traits over clinical competence, which may in turn influence interview offers and positioning on rank lists. Understanding whether these disparities reflect true differences in performance or are based on inherent evaluation biases is essential for ensuring fairness and accuracy in residency selection. 

Another key area of interest is whether repeated rotations provide any compounding benefits for the EM applicant. Previous research has produced mixed results on receiving improved grades as a result of more rotation experiences, and national advising guidelines recommend limiting the number of rotations to no more than two per applicant [12,13]. Program directors themselves have noted that there is likely little benefit to completing more than two EM rotations or obtaining additional SLOEs [14]. One study demonstrated that students who completed three “away” rotations tended to perform worse on their third rotation [15]. Research has also been conducted comparing grades of “home” vs. “away” rotations, suggesting that applicants are typically more likely to be ranked higher on their “home” rotation’s rank list [12,15]. Given the added strain in applying and completing unnecessary rotations at the expense of providing other capable applicants with this audition experience, we sought to explore any potential relationship between the SLOE grades and the number of rotators completed to provide better guidance for EM mentors.

Taken together, these questions highlight the need to examine both gender-related differences in SLOE scores and the influence of additional fourth-year rotations. Accordingly, the objective of this study was to evaluate (1) whether gender is associated with differences in SLOE scores and (2) whether performance changes across multiple EM rotations, regardless of gender.

## Materials and methods

A retrospective review was conducted of de-identified SLOEs from residents who trained between 2015 and 2021 at a large (>50 residents), three-year urban academic emergency medicine residency program in the Northeastern United States. For data analysis, all identifiable information, including applicant names, Association of American Medical Colleges (AAMC) IDs, and application years, was removed. The study received exemption from our institutional review board.

Each applicant was assigned a randomly generated study identifier, and de-identified elements such as age during rotation, letter institution, gender, and whether or not they became a chief resident were linked to each resident's SLOEs but not included in the reported analyses. Non-emergency medicine letters of evaluation, such as the Off-Service Standardized Letter of Evaluation (OSLOE), a non-EM version of the SLOE used for rotations outside of EM, or evaluations from elective rotations (e.g., two-week ultrasound elective), were excluded from the analysis. These evaluations were not considered comparable to EM SLOEs due to the lack of standardized structure, different evaluative criteria, and variable grading scales across institutions. Incomplete SLOEs, including those lacking a descriptive grade, were also excluded. The descriptive grading scales linked to each quantitative SLOE question were converted into numerical scales (Table 1) for purposes of data analysis. Because these questions were standardized single-item anchors, internal consistency reliability was not assessed. Any SLOEs lacking a descriptive grade were excluded from the analysis. For applicants rotating within the same program within a given application cycle, SLOEs were assumed to be authored by the same faculty member(s), although this was not independently verified. Of note, a small subset of SLOEs (n=10) were recorded as ‘Pass’ from institutions using pass/fail grading schemes; while retained in the analysis, these may not be directly comparable to SLOEs from graded rotations.

**Table 1:** Conversion of Descriptive SLOE Grading Scales into Numerical Values for Data Analysis ^+^10 SLOEs were passing grades from pass/fail rotations, included in the analysis as 'pass' (coded as '1'). SLOE: Standardized Letter of Evaluation

Questions	Descriptive scale	Numerical scale
1: Commitment to emergency medicine; 2: Work ethic and willingness to assume responsibility; 3: Ability to develop and justify an appropriate differential and treatment plan; 5: Ability to work in a team; 6: Ability to communicate a caring nature to patients; 9: Overall comparison of applicant to previous years' applicants; 10: Estimation of place on their rank list	Top 10%	4
	Top third	3
	Middle third	2
	Bottom third	1
4: What grade did they receive?^+^	Honors	3
	High pass	2
	Pass	1
7: How much guidance do you predict this applicant will need during residency?	Less than peers	3
	Same as peers	2
	More than peers	1
8: What is your prediction of success for this applicant?	Outstanding	3
	Excellent	2
	Good	1

Ultimately, data from 243 SLOEs (158 male, 85 female) representing 101 residents (66 male, 35 female, mean age 27.65 years) were analyzed utilizing IBM SPSS Statistics software, version 29 (IBM Corp., Armonk, NY). In EM, students typically complete multiple rotations (most often, two to four), each generating a separate SLOE; therefore, residents in this cohort contributed between one and four SLOEs. The mean number of rotations in this sample set was 2.41. Summary data statistics were calculated for the SLOEs. A two-sided Student’s t-test for independent samples assuming unequal variance was used to determine mean differences in the quantitative questions based on gender, as variability in score distributions between groups was not assumed to be equal. 

**Figure 1:**
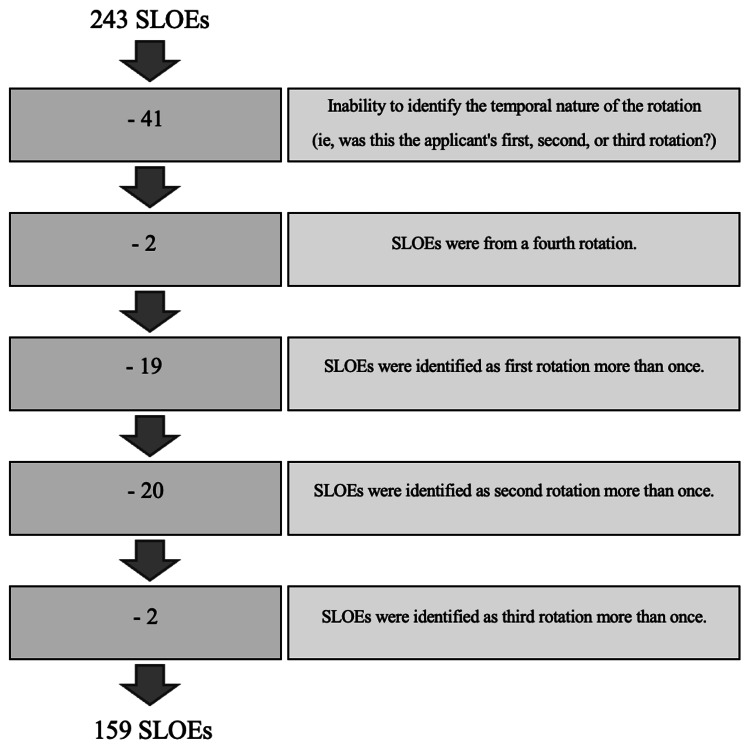
Flow Diagram of the Excluded SLOEs and Final Dataset for Rotation Order Subanalysis SLOE: Standardized Letter of Evaluation

In order to assess changes in SLOE grades across multiple fourth-year rotations, SLOEs were then also categorized based on the order in which they were completed. Out of the 243 SLOEs collected, only 159 were included in this subanalysis (Figure 1). Rotation order was determined based on the sequence indicated by the evaluator on the SLOE form. Analyses were restricted to the first three EM rotations per applicant, when available. Fourth rotations were excluded because they were uncommon and would have further limited comparability across applicants. The relatively small number of applicants with three rotations (n=29) was acknowledged as limiting statistical power in this subgroup. A one-way ANOVA test was used to determine mean differences in the quantitative questions comparing SLOEs received during the first rotation vs. second rotation vs. third rotation. 

## Results

Analysis of the grade data comparing genders revealed female applicants (35 applicants, 85 SLOEs) received higher scores compared to their male co-applicants (66 applicants, 158 SLOEs) on every question, with statistically significant differences in four out of the ten quantitative questions (Table 2). Female applicants were noted to score significantly higher on questions assessing their ability to formulate a differential and treatment plan (2.412 vs. 2.203, p = 0.008), as well as their ability to communicate a caring nature to their patients (2.635 vs. 2.481, p = 0.029). Female applicants were also noted to have higher rotation grades (2.412 vs. 2.2, p = 0.015) and higher estimated rank lists than their male counterparts (2.781 vs. 2.543, p = 0.04). No significant difference was found comparing female to male applicants in questions on commitment to emergency medicine, work ethic, and ability to work in a team. Additionally, no significant differences were found between female and male applicants in terms of their overall predicted success in residency, comparisons to previous years' applicants, and the anticipated guidance required during residency. Though trends were observed, these differences did not reach statistical significance and should be interpreted with caution.

**Table 2:** Comparison of SLOE Scores by Gender Across Quantitative Evaluation Domains A two-sided Student’s t-test for independent samples assuming unequal variance was used to determine mean differences in the quantitative questions based on gender. The totals reflect the number of SLOEs analyzed (158 males, 85 females), which exceeds the number of individual residents (66 males, 35 females) because most residents contributed multiple SLOEs. SLOEs: Standardized Letter of Evaluations

Question	Gender	Mean	n	t-test	P-value
1: Commitment to emergency medicine; has carefully thought out this career choice.	Male	2.538	158	0.044	0.966
	Female	2.541	85		
2: Work ethic, willingness to assume responsibility.	Male	2.62	158	1.422	0.154
	Female	2.718	85		
3: Ability to develop and justify an appropriate differential and cohesive treatment plan.	Male	2.203	158	2.633	0.008
	Female	2.412	85		
4: What grade did they receive?	Male	2.2	155	2.404	0.015
	Female	2.412	85		
5: Ability to work with a team.	Male	2.487	158	1.578	0.119
	Female	2.6	85		
6: Ability to communicate a caring nature to patients.	Male	2.481	158	2.241	0.029
	Female	2.635	85		
7: How much guidance do you predict this applicant will need during residency?	Male	1.835	158	0.700	0.484
	Female	1.777	85		
8: Given the necessary guidance, what is your prediction of success for the applicant?	Male	2.241	158	1.683	0.089
	Female	2.365	85		
9: Compared to other EM residency candidates you have recommended in the last academic year, this candidate is in the:	Male	2.535	157	1.649	0.107
	Female	2.718	85		
10: How highly would you estimate the candidate will reside on your rank list?	Male	2.543	151	2.123	0.04
	Female	2.781	82		

**Figure 2:**
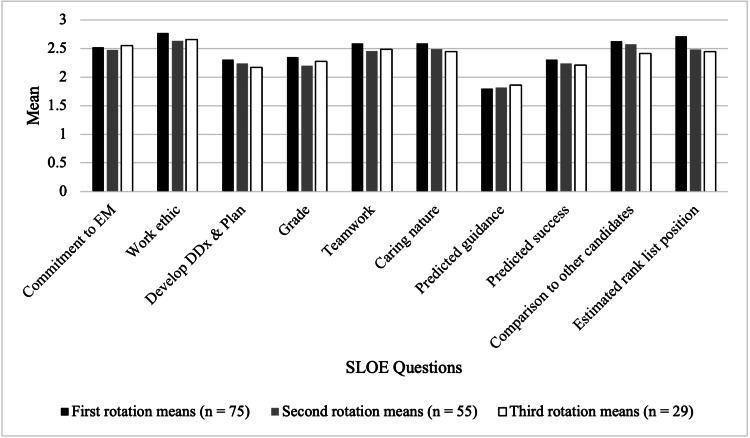
Mean Scores on SLOE Domains by Rotation Sequence Bars represent mean scores for each evaluative question across first (n = 75), second (n = 55), and third (n = 29) rotations. No statistically significant differences were observed between rotation groups. EM: Emergency Medicine; DDx: differential diagnosis; SLOE: Standardized Letter of Evaluation

A one-way ANOVA analysis of the ten quantitative questions did not reveal any statistically significant differences in grades across subsequent SLOEs (Table 3), though trends in the SLOE data did reveal several notable patterns across sub-internships (Figure 2). Commitment to EM decreased slightly during the second rotation before increasing again in the third, with the highest overall scores recorded during the third rotation (p = 0.795). Attributes such as work ethic (p = 0.217), the ability to generate a differential and make a treatment plan (p = 0.524), and the ability to communicate a caring nature to patients (p = 0.358) were rated highest during the applicants’ first rotation. Similarly, evaluators provided the most favorable ratings of applicants during their first rotation concerning their predicted future success (n = 2.307, p = 0.621), comparison to applicants from previous years (n = 2.627, p = 0.446), and estimated position on the institution’s rank list (n = 2.714, p = 0.149). While applicant grades decreased slightly during the second rotation (n = 2.2), they tended to improve during the third rotation (n = 2.276), though they still did not reach the mean grade from the first rotation (n = 2.347, p = 0.434). Finally, the amount of predicted guidance required by the applicants in residency increased numerically across subsequent rotations, indicating evaluators predicted applicants would require less guidance as they progressed through subsequent rotations (p = 0.149).

**Table 3:** Comparison of SLOE Scores Across Subsequent Sub-Internship Rotations Group comparisons across first, second, and third sub-internship rotations were conducted using one-way analysis of variance (ANOVA). Reported statistics include the F value with associated degrees of freedom and p-value. *Values reported in these columns are means with the number of SLOEs in parentheses, rather than unique residents, as residents often contributed more than one SLOE. SLOEs: Standardized Letter of Evaluations

Questions	First rotation means* (n = 75)	Second rotation means* (n = 55)	Third rotation means* (n = 29)	ANOVA F(2,156)	P value
1: Commitment to emergency medicine; has carefully thought out this career choice.	2.52	2.473	2.552	0.230	0.795
2: Work ethic, willingness to assume responsibility.	2.77	2.636	2.655	1.544	0.217
3: Ability to develop and justify an appropriate differential and cohesive treatment plan.	2.307	2.236	2.172	0.648	0.524
4: What grade did they receive?	2.347	2.2	2.276	0.840	0.434
5: Ability to work with a team.	2.587	2.455	2.483	1.102	0.335
6: Ability to communicate a caring nature to patients.	2.587	2.491	2.448	1.033	0.358
7: How much guidance do you predict this applicant will need during residency?	1.8	1.818	1.862	0.117	0.889
8: Given the necessary guidance, what is your prediction of success for the applicant?	2.307	2.236	2.207	0.477	0.621
9: Compared to other EM residency candidates you have recommended in the last academic year, this candidate is in the:	2.627	2.574	2.414	0.768	0.446
10: How highly would you estimate the candidate will reside on your rank list?	2.714	2.482	2.448	1.930	0.149

## Discussion

Our analysis revealed notable gender differences in SLOE scores. Female applicants scored higher on questions assessing their ability to formulate a differential and treatment plan, communicate a caring nature to patients, achieve higher rotation grades, and secure higher estimated rank positions. These findings align with previous research indicating that female applicants often receive higher scores in certain evaluative areas [8,9].

The lack of significant differences in questions related to commitment to EM, work ethic, and teamwork suggests that these qualities are rated similarly regardless of gender. Additionally, no significant differences were observed in overall predicted success in residency, comparisons to previous years' applicants, and the anticipated guidance required during residency. This nuanced pattern indicates that while certain domains may be susceptible to variability, others remain relatively stable across gender lines, providing some degree of reassurance regarding the objectivity of some SLOE components.

When analyzing SLOE scores across subsequent sub-internships, we found no statistically significant differences in average grades. This finding is consistent with previous studies, though it contradicts the intuitive expectation that experience leads to better evaluations [15]. One possible explanation is that students might perform better in familiar environments, and a lack of familiarity with new emergency departments could hinder their ability to demonstrate their full potential. Another likely explanation is the ‘home field advantage,’ as first rotations are frequently completed at an applicant’s home institution, where evaluators may feel implicit pressure to support their students’ match prospects and thus provide more favorable evaluations. It is also possible that higher scores in the first rotation reflect novelty bias or initial enthusiasm from evaluators when assessing students at the beginning of the application season.

Interestingly, trends in our data suggested some variations: commitment to EM decreased slightly during the second rotation but increased in the third, with the highest overall scores noted in the third rotation. However, other measures, including overall rotation grade and predicted success, were highest during the first rotation, suggesting that initial performance may leave the strongest impression. Fundamentally, this should serve as the primary focus for advising: students should not feel pressured to complete more than two SLOEs, as there is no clear statistical benefit to additional sub-internships. This supports the recent Council of Emergency Medicine Residency Directors' (CORD) guidelines that recommend limiting the number of EM sub-Is per applicant [16].

Additionally, as students progress through their sub-internships, evaluators might recognize more complex areas where guidance is necessary, leading to higher expectations and more stringent evaluations. The familiarity with the expectations and culture of a specific institution may initially facilitate higher scores, but the transition to new settings with different protocols and expectations might challenge students. This may explain why subsequent SLOE scores fail to improve and, in some cases, decline. These hypotheses remain speculative and highlight an important area for future investigation.

These findings have several implications for equity and application strategy. The opportunity to complete multiple rotations is not equally accessible to all applicants, particularly those facing financial/scheduling constraints or those without a home EM program, such as many Doctor of Osteopathic Medicine (DO) and International Medical Graduate (IMG) applicants, who may be disproportionately disadvantaged. If additional rotations do not yield higher scores, encouraging applicants to limit their rotations not only aligns with best practices but also promotes a more level playing field.

Finally, our results highlight a potential need for increased rater training and standardization across institutions. Variation in how students are scored across sites (even when performance may be consistent) suggests that faculty development on SLOE interpretation and scoring could improve inter-rater reliability and reduce unintentional bias. Future studies could explore whether gender-related differences in SLOE evaluations are associated with downstream leadership roles, such as selection as chief resident. It is also important to recognize that gender does not exist in isolation; intersecting factors such as race/ethnicity and socioeconomic background likely further shape applicant experiences and evaluations. Exploring these dimensions in future research will be essential to fully understand and address equity in the residency selection process.

Limitations

This study has several limitations. First, it was conducted at a single urban academic EM residency program, which may limit generalizability to other institutions with different evaluators, applicant pools, or grading cultures. Future research should consider multi-site data and control for these factors to enhance reproducibility across diverse settings. Institutional culture and geographic factors may also shape evaluator behavior, and similar patterns may not be observed in other settings, such as community-based or non-urban programs. Additionally, because the dataset was limited to applicants who ultimately matched at the study institution, the findings may reflect both how SLOEs were written and how they were interpreted by local reviewers during the selection process. This selection bias may further limit generalizability to the broader pool of EM applicants.

Second, the retrospective design relied on available de-identified SLOEs, and some were excluded due to incomplete data or inability to determine rotation order, which may have introduced selection bias. The number of applicants with three EM rotations was relatively small (n = 29), which reduced statistical power and may have limited the ability to detect meaningful differences in this subgroup. Third, while the SLOE is widely used in EM, it may not fully capture all dimensions of clinical competence or future success in residency. Its reliance on subjective impressions and estimated rankings introduces variability that is difficult to quantify, which could influence both individual scores and broader trends.

While gender was recorded, other demographic factors such as race/ethnicity or socioeconomic status were not available, limiting exploration of intersecting sources of bias. Additional factors that may influence SLOE ratings, such as medical school attended, prior clinical experience, or evaluator characteristics, were also not available and therefore could not be examined. Other variables collected for dataset linkage, including age and chief status, were not incorporated into subgroup analyses, as the focus of this study was on gender and number of rotations. Another limitation is that the authors were unable to account for whether SLOEs were from home versus away rotations, as this information was not consistently available in the dataset. Given prior evidence of ‘home field advantage’ in evaluation, future studies should examine whether this dynamic influences the impact of repeated rotations or contributes to gender-related differences. Also, whether the same faculty member authored all SLOEs from a given program within an application cycle was not confirmed; while a single evaluator was likely responsible, inter-rater variability remains a possible source of bias. Finally, variability in grading systems may have influenced results. The conversion of descriptive grading scales into numerical values, though necessary for analysis, may oversimplify the nuanced judgments intended by evaluators. In addition, a small subset of SLOEs (n=10) were recorded as ‘Pass’ from institutions using pass/fail grading schemes; while retained in the analysis, these may not be directly comparable to SLOEs from graded rotations.

## Conclusions

Our analysis suggests that female applicants who matched at a single, urban, academic center often receive higher scores in certain evaluative areas, and that the impact of repeated sub-internships on SLOE scores is complex and multifaceted. While SLOEs offer significant insights into the characteristics and overall potential success of residency applicants, their ability to predict specific traits and performance outcomes across genders and multiple rotations is limited. Future efforts should focus on improving standardization in SLOE evaluations and providing clear, equitable advising to ensure that all applicants are supported fairly through the residency application process.
